# Molecular characteristics and zoonotic potential of enteric protozoans in domestic small ruminants in Heilongjiang Province, Northeast China

**DOI:** 10.1016/j.fawpar.2025.e00296

**Published:** 2025-10-23

**Authors:** Meiru Hou, Xuewei Liu, Lu Zhou, Jiawang Zhou, Yuxi Zhang, Tianshuai Ma, Hongyu Qiu, Chunren Wang, Junfeng Gao

**Affiliations:** Key Laboratory of Prevention and Control of Zoonotic Diseases of Daqing, College of Animal Science and Veterinary Medicine, Heilongjiang Bayi Agricultural University, Daqing 163319, China

**Keywords:** *Cryptosporidium* spp., *Giardia duodenalis*, *Enterocytozoon bieneusi*, *Blastocystis* sp., Domestic small ruminants, Heilongjiang Province

## Abstract

*Cryptosporidium* spp., *Giardia duodenalis*, *Enterocytozoon bieneusi*, and *Blastocystis* sp. are four common zoonotic intestinal protozoa, that cause frequent foodborne and waterborne outbreaks worldwide. Despite their public health importance, epidemiological data remain scarce from Heilongjiang Province in China. Fecal samples were collected from 845 sheep and 166 goats across 13 regions of Heilongjiang Province, Northeast China. PCR-based methods were used to detect these pathogens and PCR products were sequenced to determine the species/genotypes. The overall infection rates for *Cryptosporidium* spp., *G. duodenalis*, *E. bieneusi*, and *Blastocystis* sp. were 4.15 % (42/1011), 2.67 % (27/1011), 12.15 % (127/1011), and 3.56 % (36/1011), respectively. The mixed infections with two or more protozoa occurred in 2.97 % (30/1011). The geographic location was a significant risk factor for the prevalence of *Cryptosporidium* spp., *E. bieneusi*, and *Blastocystis* sp. in domestic small ruminants. Four *Cryptosporidium* genotypes (*C. xiaoi*, *C. ubiquitum*, *C. bovis*, *C. andersoni*), seven *E. bieneusi* genotypes (BEB6, COS-I, CHS8, CHS7, CHG1, CHG3, J), two *G. duodenalis* assemblages (assemblage E, assemblage A), and six *Blastocystis* subtypes (ST10, ST14, ST26, ST5, ST15, ST30) were identified. This study provides critical data for developing control strategies with significant implications for zoonotic risk assessment in Heilongjiang Province.

## Highlights

• Domestic small ruminants act as reservoirs for four zoonotic protozoans.
• *C. xiaoi,* assemblage E, BEB6 and ST10 are dominant genotypes in four protozoans.
• Geographic location is a risk factor for zoonotic protozoans in small ruminants.

## 1 Introduction

*Cryptosporidium* spp., *Giardia duodenalis*, *Enterocytozoon bieneusi*, and *Blastocystis* sp. are common zoonotic protozoa that infect a wide range of hosts, including humans and livestock (Wang et al., 2022). These protists can be transmitted between humans and animals via the fecal–oral route or through direct contact with infected hosts (Hatam-Nahavandi et al., 2025). Infected neonatal animals may develop diarrhea, dehydration, and growth retardation, whereas adults usually remain asymptomatic but act as carriers, perpetuating infection at the herd level (Lux et al., 2023). Due to the lack of effective drugs and commercial vaccines, these protozoan diseases may result in a substantial economic impact from livestock losses (Li et al., 2025).

*Cryptosporidium* is one of the most prevalent zoonotic parasitic protozoa, infecting more than 260 animal species (Golomazou et al., 2024). It is the second most common cause of childhood diarrhea globally after rotavirus and is recognized as an opportunistic infection in immunocompromised individuals (Jiang et al., 2023). At least 47 species have been identified, with 14 species displaying the capacity to infect sheep. Among these, *C. ubiquitum*, *C. xiaoi*, *C. andersoni,* and *C. parvum* are the most common (Zhao et al., 2024). Of particular concern for public health is the presence of *C. parvum* and *C. ubiquitum* in sheep and goats (Xiao and Feng, 2017).

*G. duodenalis* is another important enteric parasite with a broad host range. It represents a species complex divided into eight assemblages (A–H) (Thompson and Ash, 2019). Assemblages A and B are the most relevant for zoonotic transmission, whereas assemblage E predominates in livestock such as sheep and goats. Although assemblages C-H were previously considered host-adapted, human infections with assemblages C, D, E, and F have been reported, suggesting broader zoonotic potential than previously assumed (Heyworth, 2016; Ryan et al., 2019). Studies of sheep and goats have identified a predominance of *G. duodenalis* assemblage E, with assemblage A occurring less frequently and assemblage B rarely detected (Louro et al., 2025).

*E. bieneusi* is a fungus-like protozoan with a global distribution among animals and humans (Nourrisson et al., 2024). More than 500 genotypes have been described, clustering into 11 phylogenetic groups (Zhang et al., 2019; Guadano-Procesi et al., 2025). Group 1 and 2 harbor most zoonotic genotypes, whereas Groups 3–11 are largely host adaptation or environment-specific (Li et al., 2019). In ruminants, genotypes from Groups 1 and 2 predominate, with genotype BEB6 frequently reported in sheep and goats in China (Wang et al., 2022). Recent molecular epidemiological surveys indicate that genotypes once considered ruminant-specific are increasingly found in humans, underscoring the importance of continued surveillance (Li et al., 2020).

*Blastocystis* sp. is one of the most common enteric protists, carried by more than one billion people worldwide (Rajamanikam et al., 2023). More than 30 subtypes (STs) have been identified based on polymorphism of the small subunit ribosomal RNA (*SSU* rRNA) gene (Hernández-Castro et al., 2023; Santin et al., 2024). In livestock, ST10 is most frequently detected, whereas in humans, ST1–ST4 predominate and account for over 95 % of *Blastocystis* sp. infections in humans. ST6–ST9 have also been detected in humans and birds, suggesting potential zoonotic links (Figueiredo et al., 2024; Piperni et al., 2024). However, the pathogenic role of *Blastocystis* sp. remains debated, and its zoonotic transmission dynamics are not yet fully understood.

Sheep and goats are crucial livestock species globally, serving as important protein sources and playing a significant role in smallholder farming systems. China has one of the world's largest populations of small ruminants, with Heilongjiang Province in Northeast China alone harboring nearly nine million sheep and goats. Previous studies have documented the presence of *Cryptosporidium* spp., *G. duodenalis*, *E. bieneusi*, and *Blastocystis* sp. in small ruminants across various regions of China (Cheng et al., 2024a; Fan et al., 2021a; Figueiredo et al., 2024). However, information on their prevalence, molecular characteristics, and zoonotic potential in Heilongjiang Province, Northeast China remains scarce. Therefore, this study aimed to investigate the prevalence and molecular characteristics of *Cryptosporidium* spp., *G. duodenalis*, *E. bieneusi*, and *Blastocystis* sp. and to assess the potential zoonotic risks in domestic small ruminants in Heilongjiang Province, Northeast China. The findings will provide baseline data for executing measures for the prevention and control of these protozoan pathogens.

## 2 Materials and methods

### 2.1 Sample collection

**Fig. 1:**
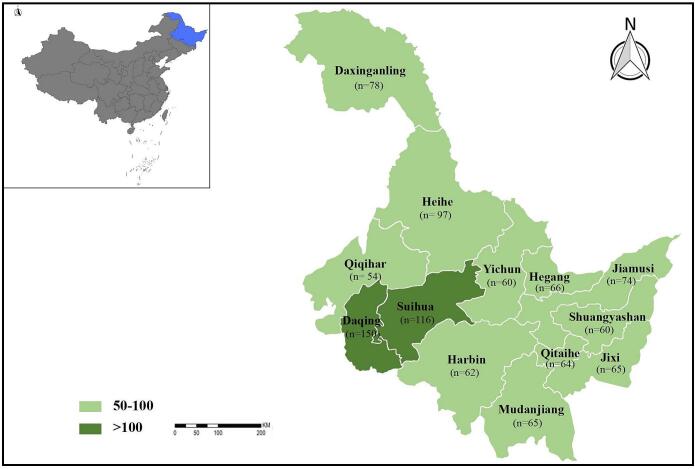
Distribution of sampling locations for domestic small ruminants in Heilongjiang Province, Northeast China.

A total of 1011 fecal samples from domestic small ruminants were collected across all 13 administrative regions of Heilongjiang Province, Northeast China between May 2023 and July 2024 (Fig. 1). The regions were selected to ensure comprehensive geographic coverage of Heilongjiang Province. Fecal samples were obtained directly by rectal collection from 845 sheep and 166 goats. The samples were stratified by sex and age to reflect proportionate distribution of livestock populations. Specifically, 686 females and 325 males were sampled, while age groups included 266 samples from animals younger than or equal to 1 year, and 745 samples from animals older than 1 year. All fecal samples were immediately placed into sterile tubes labeled with information, kept in the car refrigerator at a low temperature, and then transported to the laboratory, stored at −20 °C for detected as soon as possible.

### 2.2 DNA extraction and PCR

The genomic DNA of each fecal sample was extracted using a Solarbio Stool Genomic DNA Extraction Kit (Solarbio, Beijing, China) in accordance with the manufacturer's recommended protocol. The quality and quantity of DNA were determined by electrophoresis in a 1.5 % (*w*/*v*) agarose gel and by A260/280 measurement with a spectrophotometer (Thermo Fisher, USA), and the extracted DNA was stored at −20 °C for subsequent detection. The identification of *Cryptosporidium* spp. was performed by nested PCR amplification of the *SSU* rRNA gene (Xiao et al., 1999). The assemblage determination for *G. duodenalis* were performed by nested PCR of the *bg* gene (Lalle et al., 2005). Nested PCR was performed to detect the genotypes of *E. bieneusi* by amplifying the ITS gene locus (Buckholt et al., 2002). The *Blastocystis* sp. positive fecal samples were determined by PCR amplification the *SSU* rRNA gene (Scicluna et al., 2006). PCR reactions were performed containing 2 μL genomic DNA (for the primary PCR) or 1 μL of the first PCR amplification product (for the secondary PCR), 2.5 μL 10 *×* Ex Taq Buffer (Takara, Dalian, China), 2 μL dNTP Mixture (400 mM) (Takara, Dalian, China), 0.2 μL Ex Taq polymerase (Takara, Dalian, China), and 0.5 μL of each primer (Sangon Biotech, Shanghai, China), and added ddH_2_O up to 25 μL in a thermocycler (BioRad, USA). The amplification conditions for the first and second PCR are consistent of detection in *Cryptosporidium* spp., *G. duodenalis*, and *E. bieneusi*. The PCR reaction was executed under the following conditions: 92 °C for 5 min (initial denaturation); then 92 °C for 45 s (denaturation), 55 °C, 60 °C, 57 °C, and 57 °C for 45 s (annealing), 72 °C for 1 min (extension) for 35 cycles, and a final extension at 72 °C for 10 min (Appendix A). The secondary PCR products were considered positive when a single band of the expected size was detected in a 1.5 % (*w*/*v*) agarose gel upon ethidium-bromide staining. All positive secondary PCR products were sent to Sangon Biotech Ltd. (Shanghai, China) for sequencing from both directions.

### 2.3 Sequence and phylogenetic analysis

The obtained nucleotide sequences were assembled and edited using DNAStar version 5.0 under default parameters. Sequence identity was confirmed by comparison with reference sequences available in GenBank using BLAST (http://www.ncbi.nlm.nih.gov/blast/). For genotype and subtype determination of *Cryptosporidium* spp., *G. duodenalis*, *E. bieneusi*, and *Blastocystis* sp., multiple sequence alignments were performed using Clustal X version 1.83 (http://www.clustal.org) with default gap opening and extension penalties to ensure accurate codon alignment. Phylogenetic trees were reconstructed using the maximum likelihood (ML) method implemented in MEGA version 11.0. The best-fit nucleotide substitution model for each dataset was selected automatically by MEGA based on the lowest Bayesian Information Criterion (BIC) score. Rate variation among sites was modeled with a gamma distribution, and the reliability of the tree topologies was assessed by bootstrap analysis with 1000 pseudoreplicates. The phylograms were visualized and annotated using FigTree version 1.4.4 (http://tree.bio.ed.ac.uk/software/figtree/). Only bootstrap values ≥50 % were displayed on the consensus trees.

### 2.4 Statistical analysis

All statistical analyses were conducted using SPSS version 22.0. The prevalence of each protozoan was calculated with corresponding 95 % confidence interval (CI) using the Wilson method. Associations between infection status and categorical variables such as host species, sex, and age group were assessed using the Chi-square test. Odds ratio (OR) with 95 % CI was calculated to estimate the strength of associations. A *p*-value of <0.05 was considered statistically significant.

## 3 Results

### 3.1 Prevalence and species distribution of Cryptosporidium spp. in domestic small ruminants

**Fig. 2:**
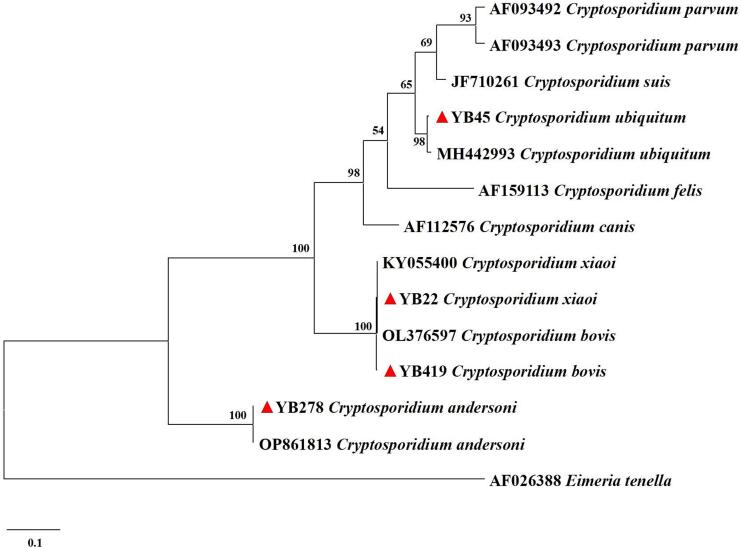
Phylogenetic analysis of *Cryptosporidium* spp. from domestic small ruminants in Heilongjiang Province, Northeast China based on the maximum likelihood method. Representative sequences obtained from this study are indicated with red triangles. Bootstrap value higher than 50 % is shown. (For interpretation of the references to colour in this figure legend, the reader is referred to the web version of this article.)

*Cryptosporidium* spp. was detected in 42 out of the 1011 samples collected from 13 administrative regions of Heilongjiang Province, with a prevalence of 4.2 % (95 % CI: 2.9–5.4) (Table 1). The highest prevalence was observed in Daqing City (17.3 %, 95 % CI: 11.3–23.4; 26/150), followed by Mudanjiang City (12.3 %, 95 % CI: 4.3–20.3; 8/65), and Qiqihar City (7.4 %, 95 % CI: 0.4–14.4; 4/54). However, no infections were obtained in Harbin, Hegang, Heihe, Jixi, Jiamusi, Suihua, or Yichun. The prevalence differed significantly among the 13 regions (*p* < 0.001). By host type groups, the prevalence was 3.3 % in sheep (95 % CI: 2.1–4.5; 28/845) and 8.4 % in goats (95 % CI: 4.2–12.7; 14/166), with a significantly higher risk in goats (*p* = 0.003). By age groups, the prevalence in younger than or equal to 1 year (13.1 %, 95 % CI: 9.1–17.2; 35/266) was markedly higher than those in old than 1 year (0.9 %, 95 % CI: 0.2–1.6; 7/745), and the difference was highly significant (p < 0.001). By gender groups, the prevalence was 3.5 % in females (95 % CI: 2.1–4.9; 24/686) and 5.5 % in males (95 % CI: 3.1–8.0; 18/325), but this difference was not statistically significant (*p* = 0.129). In this study, phylogenetic analysis was conducted for 42 sequenced samples of *Cryptosporidium* utilizing the ML method (Fig. 2). Four known species were identified, namely *C. andersoni* (0.1 %, 1/1011), *C. bovis* (0.3 %, 3/1011), *C. ubiquitum* (0.6 %, 6/1011), and *C. xiaoi* (3.2 %, 32/1011). Among these, *C. xiaoi* was the dominant species and was detected in all regions with *Cryptosporidium* spp. infection except Qitaihe City. *C. xiaoi* was also detected across different host type, gender and age groups. At the same time, only one *C. andersoni* positive sample was detected, from a female goat aged younger than or equal to 1 year in Daxinganling Prefecture. Importantly, the zoonotic species *C. ubiquitum* was identified in domestic small ruminants, suggesting a potential risk for human infection through direct contact with infected animals or contaminated environments.

**Table 1:** Prevalence and species distribution of *Cryptosporidium* spp. in domestic small ruminants in Heilongjiang Province, Northeast China.

Variable	No. of sample	No. of Positive	Prevalence% (95 % CI)	OR (95 % CI)	*p*-Vale	Species (No.)
Region						
Daqing	150	26	17.3 (11.3–23.4)	13.2 (1.8–99.6)	< 0.001	*C. bovis* (2), *C. ubiquitum* (4)*, C. xiaoi* (20)
Daxinganling	78	2	2.6 (0.0–6.1)	1.7 (0.2–18.7)		*C. andersoni* (1), *C. xiaoi* (1)
Harbin	62	0	–	–		–
Hegang	66	0	–	–		–
Heihe	97	0	–	–		–
Jixi	65	0	–	–		–
Jiamusi	74	0	–	–		–
Mudanjiang	65	8	12.3 (4.3–20.3)	8.8 (1.1–72.9)		*C. bovis* (1), *C. xiaoi* (7)
Qitaihe	64	1	1.6 (0.0–4.6)	1		*C. ubiquitum* (1)
Qiqihar	54	4	7.4 (0.4–14.4)	5.0 (0.6–46.5)		*C. ubiquitum* (1), *C. xiaoi* (3)
Shuangyashan	60	1	1.7 (0.0–4.9)	1.1 (0.1–17.5)		*C. xiaoi* (1)
Suihua	116	0	–	–		–
Yichun	60	0	–	–		–

Type						
Sheep	845	28	3.3 (2.1–4.5)	1	0.003	*C. bovis* (2), *C. ubiquitum* (4), *C. xiaoi* (22)
Goat	166	14	8.4 (4.2–12.7)	2.7(1.4–5.2)		*C. andersoni* (1), *C. bovis* (1), *C. ubiquitum* (2), *C. xiaoi* (10)

Gender						
Female	686	24	3.5 (2.1–4.9)	1	0.129	*C. andersoni* (1), *C. bovis* (3), *C. ubiquitum* (3), *C. xiaoi* (17)
Male	325	18	5.5 (3.1–8.0)	1.6 (0.9–3.0)		*C. andersoni* (3), *C. xiaoi* (15)

Age						
≤ 1 Year	266	35	13.1 (9.1–17.2)	16.0 (7.0–36.5)	< 0.001	*C. andersoni* (1), *C. bovis* (3), *C. ubiquitum* (4), *C. xiaoi* (27)
>1 Year	745	7	0.9 (0.2–1.6)	1		*C. ubiquitum* (2), *C. xiaoi* (5)
Total	1011	42	4.2 (2.9–5.4)			*C. andersoni* (1), *C. bovis* (3), *C. ubiquitum* (6), *C. xiaoi* (32)

### 3.2 Prevalence and species distribution of G. duodenalis in domestic small ruminants

**Fig. 3:**
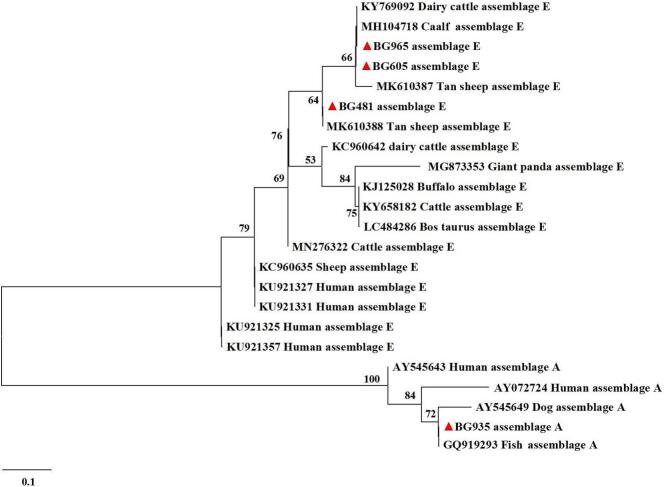
Phylogenetic analysis of *Giardia duodenalis* from domestic small ruminants in Heilongjiang Province, Northeast China based on the maximum likelihood method. Representative sequences obtained from this study are indicated with red triangles. Bootstrap value higher than 50 % is shown. (For interpretation of the references to colour in this figure legend, the reader is referred to the web version of this article.)

Regarding *G. duodenalis*, a total of 27 positive samples were detected by nested PCR amplification of the *bg* gene, representing a 2.7 % infection rate (95 % CI: 1.7–3.7; Table 2). The highest prevalence was recorded in Hegang City (6.1 %, 95 % CI: 0.3–11.8; 4/66), followed by Jiamusi City (5.4 %, 95 % CI: 0.3–10.6; 4/74), and Harbin City (4.8 %, 95 % CI: 0.0–10.2; 3/62). *G. duodenalis* was not detected in Jixi, Mudanjiang, Suihua or Yichun. The differences in prevalence rates among the 13 regions were not statistically significant (*p* = 0.268). By host type groups, the prevalence was 2.6 % in sheep (95 % CI: 1.5–3.7; 22/845) and 3.0 in goats (95 % CI: 0.4–5.6; 5/166), with no significant difference (*p* = 0.765). By gender groups, the prevalence was 2.5 % in females (95 % CI: 1.3–3.6; 17/686) and 3.1 % in males (95 % CI: 1.2–5.0; 10/325), with no significant difference (*p* = 0.581). By age groups, the prevalence in younger than or equal to 1 year was 2.3 % (95 % CI: 0.5–4.0; 6/266), while those in older than 1 year was 2.8 % (95 % CI: 1.6–4.0; 21/745). Similarly, the differences were not statistically significant (*p* = 0.625). A total of 27 *G. duodenalis*-positive samples were identified by comparative analysis with the *bg* gene, revealing two known genotypes: assemblage E and assemblage A (Fig. 3). The predominant genotype was assemblage E (*n* = 26), which was distributed across different region, host type, gender, and age groups. Only one assemblage A sample was detected from a female sheep aged older than 1 year in Heihe City.

**Table 2:** Prevalence and species distribution of *G. duodenalis* in domestic small ruminants in Heilongjiang Province, Northeast China.

Variable	No. of sample	No. of Positive	Prevalence% (95 % CI)	OR (95 % CI)	*p*-Vale	Assemblages (No.)
Region						
Daqing	150	7	4.7 (1.3–8.0)	3.8 (0.5–31.2)	0.268	E (7)
Daxinganling	78	1	1.3 (0.0–3.8)	1		E (1)
Harbin	62	3	4.8 (0.0–10.2)	3.9 (0.4–38.6)		E (3)
Hegang	66	4	6.1 (0.3–11.8)	5.0 (0.5–45.6)		E (4)
Heihe	97	3	3.1 (0.0–6.5)	2.5 (0.3–24.1)		A (1), E (2)
Jixi	65	0	–	–		–
Jiamusi	74	4	5.4 (0.3–10.6)	4.4 (0.5–40.3)		E (4)
Mudanjiang	65	0	–	–		–
Qitaihe	64	3	4.7 (0.0–9.9)	3.8 (0.4–37.3)		E (3)
Qiqihar	54	1	1.9 (0.0–5.4)	1.5 (0.1–23.7)		E (1)
Shuangyashan	60	1	1.7 (0.0–4.9)	1.3 (0.1–21.3)		E (1)
Suihua	116	0	–	–		–
Yichun	60	0	–	–		–
Type						
Sheep	845	22	2.6 (1.5–3.7)	1	0.765	E (21), A (1)
Goat	166	5	3.0 (0.4–5.6)	1.2 (0.4–3.1)		E (5)

Gender						
Female	686	17	2.5 (1.3–3.6)	1	0.581	E (16), A (1)
Male	325	10	3.1 (1.2–5.0)	1.3 (0.6–2.8)		E (10)

Age						
≤ 1 Year	266	6	2.3 (0.5–4.0)	1	0.625	E (6)
>1 Year	745	21	2.8 (1.6–4.0)	3.0 (1.0–8.8)		E (20), A (1)
Total	1011	27	2.7 (1.7–3.7)	2.1 (0.2–15.8)		E (26), A (1)

### 3.3 Prevalence and species distribution of E. bieneusi in domestic small ruminants

**Fig. 4:**
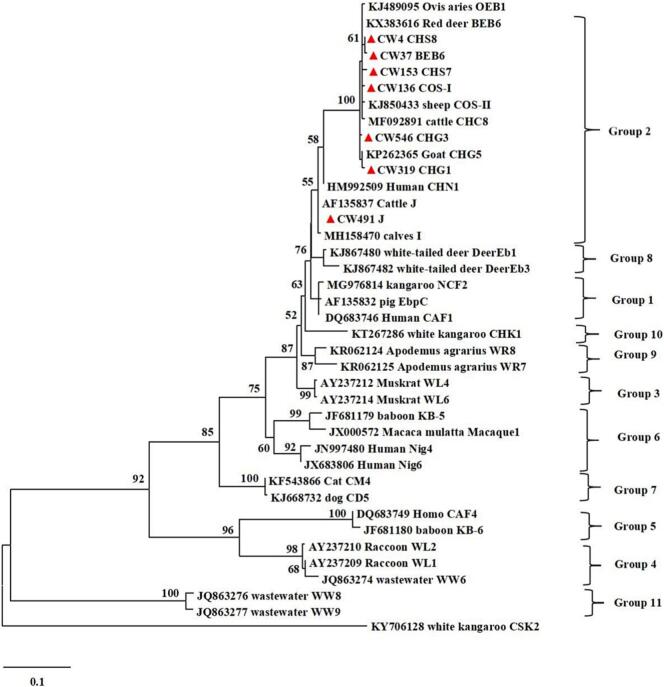
Phylogenetic analysis of *Enterocytozoon bieneusi* from domestic small ruminants in Heilongjiang Province, Northeast China based on the maximum likelihood method. Representative sequences obtained from this study are indicated with red triangles. Bootstrap value higher than 50 % is shown. (For interpretation of the references to colour in this figure legend, the reader is referred to the web version of this article.)

Regarding *E. bieneusi*, a total of 127 positive samples were detected by nested PCR amplification of the ITS gene, representing an infection rate of 12.2 % (95 % CI: 10.5–14.6; Table 3). The highest prevalence was recorded in Suihua City (37.9 %, 95 % CI: 29.1–46.8; 44/116), followed by Daqing City (26.0 %, 95 % CI: 19.0–33.0; 39/150), and Jiamusi City (16.2 %, 95 % CI: 7.8–24.6; 12/74). *E. bieneusi* was not detected in Jixi or Mudanjiang. The prevalence differed significantly among the 13 regions (*p* < 0.001). By gender groups, males showed a higher prevalence (16.6 %, 95 % CI: 12.6–20.7; 54/325) compared to females (10.6 %, 95 % CI: 8.3–12.9; 73/686), and the difference was statistically significant (*p* = 0.007). By host type groups, the prevalence in sheep was 12.8 % (95 % CI: 10.5–15.0; 108/845) and in goats 11.5 % (95 % CI: 6.6–16.3; 19/166), showing no significant difference (*p* = 0.635). By age groups, the prevalence in younger than or equal to 1 year was 15.4 % **(**95 % CI: 11.1–19.8; 41/266), while those in old than 1 year was 11.5 % (95 % CI: 9.2–13.8; 86/745), the difference was not statistically significant (*p* = 0.102). In present study, 127 *E. bieneusi*-positive samples were identified as seven known genotypes by comparative analysis of ITS gene sequences, namely COS-I, BEB6, CHG1, CHG3, CHS7, CHS8, and J (Fig. 4). Of these, the most common genotype was BEB6 (*n* = 67), followed by the COS-I genotype (*n* = 34). Only one genotype J positive sample was obtained, from a female sheep aged younger than or equal to 1 year in Harbin City. Phylogenetic analysis indicated that all the genotypes obtained in the present study belonged to Group 2.

**Table 3:** Prevalence and species distribution of *E. bieneusi* in domestic small ruminants in Heilongjiang Province, Northeast China.

Variable	No. of sample	No. of ositive	Prevalence% (95 % CI)	OR (95 % CI)	*p*-Vale	Genotypes (No.)
Region						
Daqing	150	39	26.0 (19.0–33.0)	33.7 (4.6–250.1)	< 0.001	BEB6 (32), COS-I (3), CHS8 (4)
Daxinganling	78	7	9.0 (2.6–15.3)	9.5 (1.1–78.7)		CHS8 (1), COS-I (1), CHG1 (5)
Harbin	62	4	6.5 (0.3–12.6)	6.6 (0.7–60.7)		J (1), BEB6 (3)
Hegang	66	7	10.6 (3.2–18.0)	11.4 (1.4–94.9)		BEB6 (5), COS-I (1), CHS7 (1)
Heihe	97	1	1.0 (0.0–3.0)	1		CHS7 (1)
Jixi	65	0	–	–		–
Jiamusi	74	12	16.2 (7.8–24.6)	18.6 (2.4–146.5)		BEB6 (7), CHG3 (2), COS-I (1), CHS8 (2)
Mudanjiang	65	0	–	–		–
Qitaihe	64	2	3.1 (0.0–7.4)	3.1 (0.3–34.9)		CHS7 (2)
Qiqihar	54	5	9.3 (1.5–1.7)	9.8 (1.1–86.2)		BEB6 (2), CHG3 (3)
Shuangyashan	60	4	6.7 (0.4–13.0)	6.9 (0.8–62.9)		BEB6 (3), COS-I (1)
Suihua	116	44	37.9 (29.1–46.8)	36.4 (4.9–269.2)		COS-I (27), BEB6 (13), CHS7 (3), CHS8 (1)
Yichun	60	2	3.3 (0.0–7.9)	3.3 (0.3–37.3)		BEB6 (2)

Type						
Sheep	845	108	12.8 (10.5–15.0)	1.1 (0.7–1.9)	0.635	COS-I (32), BEB6 (56), CHG1 (3), CHG3 (2), CHS7 (7), CHS8 (7), J (1)
Goat	166	19	11.5 (6.6–16.3)	1		COS-I (2), BEB6 (11), CHG1 (2), CHG3 (3), CHS8 (1)

Gender						
Female	686	73	10.6 (8.3–12.9)	1	0.007	COS-I (21), BEB6 (33), CHG1 (5), CHG3 (4), CHS7 (5), CHS8 (4), J (1)
Male	325	54	16.6 (12.6–20.7)	1.7 (1.2–2.5)		BEB6 (34), CHG3 (1), CHS7 (2), CHS8 (4), COS-I (13)

Age						
≤ 1 Year	266	41	15.4 (11.1–19.8)	1.4 (0.9–2.1)	0.102	BEB6 (26), CHG1 (5), CHG3 (2), CHS8 (3), COS-I (4), J (1)
>1 Year	745	86	11.5 (9.2–13.8)	1		BEB6 (41), COS-I (30), CHG3 (3), CHS7 (7), CHS8 (5)
Total	1011	127	12.2 (10.5–14.6)			COS-I (34), BEB6 (67), CHG1 (5), CHG3 (5), CHS7 (7), CHS8 (8), J (1)

### 3.4 Prevalence and species distribution of Blastocystis sp. in domestic small ruminants

**Fig. 5:**
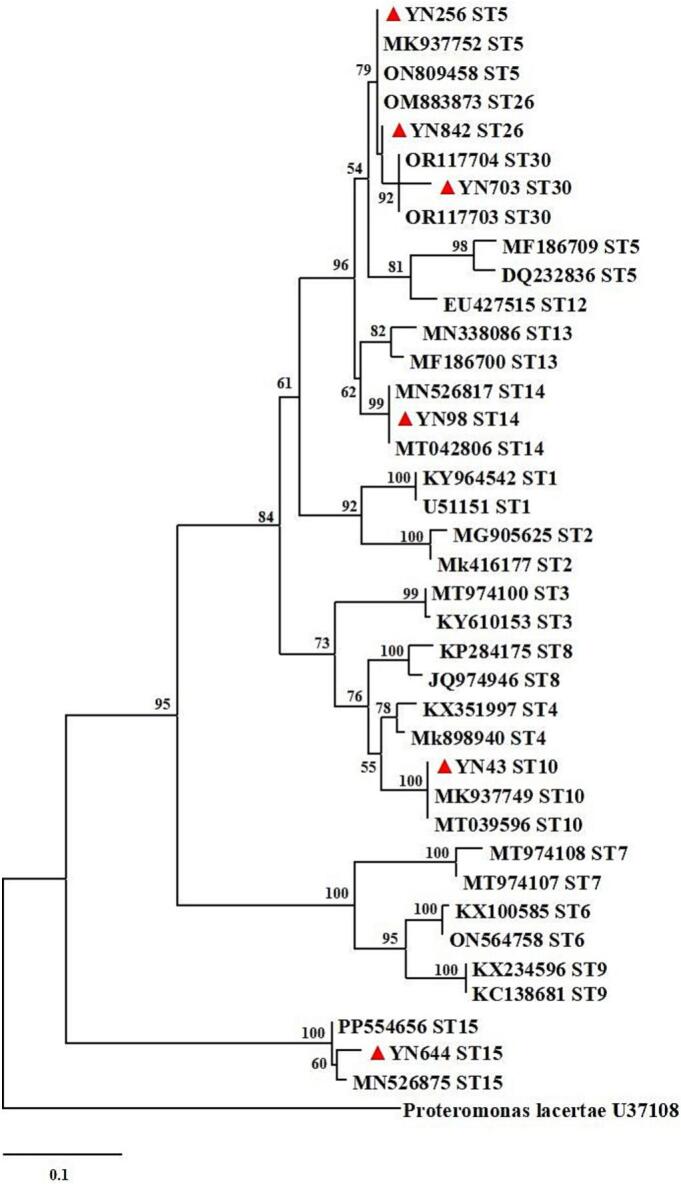
Phylogenetic analysis of *Blastocystis* sp. from domestic small ruminants in Heilongjiang Province, Northeast China based on the maximum likelihood method. Representative sequences obtained from this study are indicated with red triangles. Bootstrap value higher than 50 % is shown. (For interpretation of the references to colour in this figure legend, the reader is referred to the web version of this article.)

A total of 1011 fecal samples collected from domestic small ruminants in Heilongjiang Province were tested, and 36 *Blastocystis*-positive samples were detected by nested PCR amplification of the *SSU* rRNA gene, representing an infection rate of 3.6 % (95 % CI: 2.4–4.7; Table 4). The highest prevalence was detected in Daqing City (8.7 %, 95 % CI: 4.2–13.2; 13/150), followed by Daxinganling Prefecture (6.4 %, 95 % CI: 1.0–11.8; 5/78), and Jiamusi City (5.4 %, 95 % CI: 0.3–10.6; 4/74). *Blastocystis* sp. was not detected in Heihe, Jixi or Mudanjiang. The prevalence differed significantly among the 13 regions (*p* < 0.05). By host type groups, the prevalence was 3.3 % in sheep (95 % CI: 2.1–4.5; 28/845) and 4.8 % in goats (95 % CI: 1.6–8.1; 8/166), with no statistically significant difference (*p* = 0.765). By gender groups, the prevalence was 2.8 % in females (95 % CI: 1.5–4.0; 19/686) and 5.2 % in males (95 % CI: 2.8–7.7; 17/325), with no statistically significant difference (*p* = 0.581). By age groups, the prevalence in younger than or equal to 1 year was 6.8 % (95 % CI: 3.7–9.8; 18/266), while in old than 1 year was 2.4 % (95 % CI: 1.3–3.5; 18/745). Likewise, the difference was not statistically significant (*p* = 0.625). In the present study, 36 *Blastocystis*-positive samples were identified as six known subtypes base on *SSU* rRNA gene sequences, namely ST5, ST10, ST14, ST15, ST26, and ST30 (Fig. 5). Among these, the most frequent subtype was ST10 (*n* = 22), followed by ST14 (*n* = 8). Only one ST5 subtype sample was obtained from a female sheep aged older than 1 year in Jiamusi City. Similarly, only one ST15 subtype sample was obtained from a female goat aged older than 1 year in Qitaihe City, and one ST30 subtype sample from a male sheep aged older than 1 year in Hegang City.

**Table 4:** Prevalence and species distribution of *Blastocystis* sp. in domestic small ruminants in Heilongjiang Province, Northeast China.

Variable	No. of sample	No. of Positive	Prevalence% (95 % CI)	OR (95 % CI)	*p*-Vale	Genotypes (No.)
Region						
Daqing	150	13	8.7 (4.2–13.2)	6.0 (0.8–46.7)	< 0.05	ST10 (12), ST14 (1)
Daxinganling	78	5	6.4 (1.0–11.8)	4.3 (0.5–37.9)		ST10 (4), ST14 (1)
Harbin	62	3	4.8 (0.0–10.2)	3.2 (0.3–31.7)		ST10 (2), ST14 (1)
Hegang	66	2	3.0 (0.0–7.2)	2.0 (0.2–22.3)		ST26 (1), ST30 (1)
Heihe	97	0	–	–		–
Jixi	65	0	–	–		–
Jiamusi	74	4	5.4 (0.3–10.6)	3.6 (0.4–33.1)		ST10 (3), ST5 (1)
Mudanjiang	65	0	–	–		–
Qitaihe	64	1	1.6 (0.0–4.6)	1		ST15 (1)
Qiqihar	54	1	1.9 (0.0–5.4)	1.2 (0.1–19.5)		ST14 (1)
Shuangyashan	60	1	1.7 (0.0–4.9)	1.1 (0.1–17.5)		ST26 (1)
Suihua	116	5	4.3 (0.6–8.0)	2.8 (0.3–24.8)		ST10 (1), ST14 (4)
Yichun	60	1	1.7 (0.0–4.9)	1.1 (0.1–17.5)		ST26 (1)

Type						
Sheep	845	28	3.3 (2.1–4.5)	1	0.765	ST10 (18), ST14 (6), ST26 (2), ST5 (1), ST30 (1)
Goat	166	8	4.8 (1.6–8.1)	1.5 (0.7–3.3)		ST10 (4), ST14 (2), ST26 (1), ST15 (1)

Gender						
Female	686	19	2.8 (1.5–4.0)	1	0.581	ST10 (12), ST14 (4), ST26 (1), ST5 (1), ST15 (1)
Male	325	17	5.2 (2.8–7.7)	1.9 (1.0–3.8)		ST10 (10), ST14 (4), ST26 (2), ST30 (1)

Age						
≤ 1 Year	266	18	6.8 (3.7–9.8)	2.9 (1.5–5.7)	0.625	ST10 (15), ST14 (3)
>1 Year	745	18	2.4 (1.3–3.5)	1		ST10 (7), ST14 (5), ST26 (3), ST5 (1), ST15 (1), ST30 (1)
Total	1011	36	3.6 (2.4–4.7)			ST5 (1), ST10 (22), ST14 (8), ST15 (1), ST26 (3), ST30 (1)

### 3.5 Co-infection of four enteric protozoans in domestic small ruminants

Co-infection with multiple protozoa was identified in 3.0 % (30/1011) of fecal samples, comprising 14 from goats and 16 from sheep. Among these, two samples showed triple infections with *Cryptosporidium* spp., *E. bieneusi* and *Blastocystis* sp., while 28 samples exhibited dual infections, including nine samples with *E. bieneusi* and *Blastocystis* sp., seven samples with *Cryptosporidium* spp. and *E. bieneusi*, seven samples with *Cryptosporidium* spp. and *Blastocystis* sp., and five samples with *G. duodenalis* and *E. bieneusi* (Appendix B). Overall, co-infections were predominantly dual infections, with combinations involving *E. bieneusi* accounting for most cases.

## 4 Discussion

*Cryptosporidium* spp. are important zoonotic intestinal protozoa that cause intestinal diseases in humans and animals (Golomazou et al., 2024). In the present study, the overall infection rate of *Cryptosporidium* spp. in domestic small ruminants in Heilongjiang Province was 4.2 %, with prevalences of 3.3 % and 8.4 % in sheep and goats, respectively. For sheep, the infection rate in the present study is similar to that reported for Qinghai Province (3.68 %, 28/761) (Yang et al., 2022) and Henan Province (4.8 %, 82/1701) (Wang et al., 2010), but higher than the rates previously reported for Jiangsu Province (1.09 %, 3/274) (Cheng et al., 2024b), Inner Mongolia Autonomous Region (1.4 %, 7/491) (Lang et al., 2023), and lower than those reported for Xinjiang Uyghur Autonomous Region (8.0 %, 100/1252) (Wang et al., 2023), and Yunnan Province (8.1 %, 23/285) (Zhang et al., 2020a). For goats, the infection rate in the present study is higher than that previously reported for Inner Mongolia Autonomous Region (3.0 %, 16/541) (Lang et al., 2023), Gansu Province (4.5 %, 8/177) (Wu et al., 2018), and Sichuan Province (4.7 %, 16/342) (Zhong et al., 2018), but lower than those reported for Shaanxi Province (16.5 %, 104/629) (Peng et al., 2016), and Yunnan Province (18.0 %, 55/305) (Zhang et al., 2020b). From a global perspective, the overall prevalence in the present study is similar to that reported from Papua New Guinea (3.2 %, 16/504) (Koinari et al., 2014) and Turkey (3.6 %, 18/500) (Aslan Çelik et al., 2023), but lower than those reported by most previous studies, such as Italy (10.1 %, 92/915) (Dessì et al., 2020), Algeria (11.0 %, 17/154) (Baroudi et al., 2018), Portugal (12.7 %, 8/63) (Gomes-Gonçalves et al., 2023), and Norway (17.0 %, 66/394) (Kifleyohannes et al., 2025). These findings indicate that *Cryptosporidium* spp. are prevalent in goats and sheep in China, with infection levels varying by region and broadly aligning with the lower end of the range reported internationally.

Our study revealed significant regional variations in *Cryptosporidium* spp. infection among sheep and goats across Heilongjiang province. The pathogen was detected in 6 of 13 surveyed regions, with Daqing City showing the highest prevalence rate (17.3 %, 26/150). Notably, animals in age younger than 1 year demonstrated significantly higher infection rates (13.1 %, 35/266) than those in age older than 1 year (0.9 %, 7/745), which is similar to previous findings indicating higher prevalence in lambs than adult animals worldwide (Chen et al., 2022). This phenomenon was also observed in Inner Mongolia Autonomous Region, where higher prevalence was reported in 15–16-week-old weaned lambs than in 3–4-week-old pre-weaned lambs (Ye et al., 2013). This higher prevalence in young animals may be attributed to their weakened immunity, weaning stress, and the prolonged incubation period of *Cryptosporidium* spp., which facilitates infection from oocysts shed by adult sheep or even through vertical transmission. Molecular characterization identified four *Cryptosporidium* species circulating in the study area. *C. xiaoi* (*n* = 32) emerged as the predominant species, being detected in nearly all positive farms and across different groups. These findings are broadly consistent with previous reports in climatically similar provinces, such as Inner Mongolia Autonomous Region, Xinjiang Uyghur Autonomous Region, and Qinghai Province (Lang et al., 2023; Wang et al., 2023; Yang et al., 2022). Notably, *C. xiaoi* (previously classified as the *C. bovis*-like genotype) has been recognized as the most prevalent *Cryptosporidium* species in domestic small ruminants in most regions except Europe (Fan et al., 2021b), and appears particularly well-adapted to sheep and goats in China (Feng and Xiao, 2017). Of particular public-health relevance was the detection of *C. ubiquitum*, a zoonotic *Cryptosporidium* species known to infect humans. This finding highlights the potential for cross-species transmission and underscores the need for enhanced, integrated surveillance in animal and human populations. The identification of *C. ubiquitum* in domestic small ruminants suggests these hosts may contribute to the local reservoir for zoonotic transmission to humans in Heilongjiang Province. Future studies should integrate expanded animal sampling, environmental monitoring, and high-resolution genotyping to clarify transmission dynamics and inform targeted control measures.

*G. duodenalis* has been recognized as a significant intestinal protozoan pathogen with global distribution in sheep and goats (Adam, 2021). Our study revealed an overall 2.7 % infection rate among domestic small ruminants in Heilongjiang Province, with sheep and goats showing similar prevalences of 2.6 % and 3.0 %, respectively. For sheep, the infection rate of *G. duodenalis* in the present study is similar to that reported for Inner Mongolia Autonomous Region (3.4 %, 27/797) (Fu et al., 2022), but higher than that reported for Gansu Province (1.7 %, 3/177) (Wu et al., 2018), Qinghai Province (1.58 %, 12/761) (Yang et al., 2022), Tibet Autonomous Region (0.8 %, 5/620) (Chang et al., 2020), and lower than that reported for Yunnan Province (21.8 %, 71/325) (Chen et al., 2019), Ningxia Hui Autonomous Region (14.5 %, 147/1014) (Peng et al., 2020), Xinjiang Uyghur Autonomous Region (7.5 %, 24/318) (Qi et al., 2019), and Henan Province (6.65 %, 47/716) (Wang et al., 2016). For goats, the infection rate of *G. duodenalis* in the present study is similar to that reported for Inner Mongolia Autonomous Region (3.7 %, 21/561) (Fu et al., 2022), but lower than that reported for Sichuan Province (14.9 %, 51/342) (Zhong et al., 2018), and Yunnan Province (4.8 %, 16/336) (Chen et al., 2019). From a global perspective, the overall prevalence in the present study is similar to that reported from Ethiopia (2.6 %, 10/389) (Wegayehu et al., 2017) and Italy (1.5 %, 5/325) (Giangaspero et al., 2005), but lower than those reported by most previous studies, such as Iran (5.2 %, 25/484) (Kiani-Salmi et al., 2019), Turkey (10.2 %, 51/500) (Aslan Çelik et al., 2023), Australia (11.1 %, 53/477) (Yang et al., 2009), and Norway (16.0 %, 61/394) (Kifleyohannes et al., 2025). Notably, Heilongjiang Province prevalence rates were 5.6 % (30/539) in sheep and 2.9 % (4/139) in goats in a study reported in 2012 (Zhang et al., 2012), which are higher than the infection rates in present study. The observed prevalence decline over the past decade may result from improved intensive farming practices and better herd management strategies that reduce environmental contamination. Furthermore, enhanced parasite control measures, particularly the adoption of regular deworming protocols, may have also played a significant role. These combined factors have likely contributed to the reduced transmission dynamics observed in the current study.

To date, eight assemblages (A–H) of *G. duodenalis* have been characterized across various host species. Among these, assemblage E demonstrates strong host specificity, predominantly infecting cloven-hoofed livestock, including cattle, sheep, goats, and pigs, although cross-species transmission to other domestic animals and non-human primates has been documented (Wang et al., 2022). Our study identified assemblage E (*n* = 26) as the predominant genotype in domestic small ruminants in Heilongjiang Province, consistent with epidemiological reports from climatically similar provinces, including Inner Mongolia Autonomous Region and Xinjiang Uyghur Autonomous Region (Fu et al., 2022; Qi et al., 2019). Intriguingly, recent molecular evidence suggests potential zoonotic capacity in some assemblage E strains (Zahedi et al., 2017). Current genotyping data confirm that assemblages A and B demonstrate the broadest host range and represent the principal genotypes associated with human giardiasis. Of particular significance was the detection of assemblage A (*n* = 1), the predominant zoonotic genotype known to infect humans, in the present study. These findings suggest that both assemblage A and E may pose zoonotic risks, indicating that infected animals could serve as reservoir hosts for potential anthroponotic transmission.

*E. bieneusi* has been recognized as a significant fungal pathogen causing intestinal microsporidiosis in domestic small ruminants worldwide, with particular public health importance due to its zoonotic potential (Guadano-Procesi et al., 2025). In the present study, the infection rate of *E. bieneusi* in Heilongjiang Province was 12.2 %, with prevalence rates of 12.8 % in sheep and 11.5 % in goats. For sheep, the *E. bieneusi* infection rate for Heilongjiang Province determined in the present study is similar to the reported rates for Yunnan Province (12.31 %, 40/325) (Chen et al., 2018), Tibet Autonomous Region (15 %, 93/620) (Chang et al., 2020), but higher than those reported for Qinghai Province (6.44 %, 49/761) (Yang et al., 2022), Xinjiang Uyghur Autonomous Region (6.3 %, 20/318) (Qi et al., 2019), Inner Mongolia Autonomous Region (4.3 %, 16/375) (Ye et al., 2015), and lower than those reported for Ningxia Hui Autonomous Region (41.1 %, 148/360) (Zhang et al., 2020b), Jiangsu Province (38.89 %, 77/198) (Cheng et al., 2024b), and Gansu Province (34.5 %, 61/177) (Wu et al., 2018). For goats, the *E. bieneusi* infection rate for Heilongjiang Province determined in the present study is higher than those reported for Tibet (9.6 %, 25/260) (Chang et al., 2020), Yunnan Province (8.9 %, 30/336) (Chen et al., 2018), but lower than those reported for Jiangsu Province (35.54 %, 209/588) (Cheng et al., 2024a), and Ningxia Hui Autonomous Region (29.7 %, 89/300) (Zhang et al., 2020a). From a global perspective, the overall prevalence in the present study is similar to those reported from Iran (18.5 %, 37/200) (Hatam-Nahavandi et al., 2025) and Brazil (19.2 %, 24/125) (Fiuza et al., 2016), but higher than that reported for Portugal (6.4 %, 4/63) (Gomes-Gonçalves et al., 2023), and lower than that reported for Sweden (68.0 %, 49/72) (Stensvold et al., 2014). Notably, Heilongjiang Province reported earlier infection rates of 22.5 % (31/138) in sheep and 21.8 % (12/55) in goats (Zhao et al., 2015), which are higher than the infection rates in the present study. The observed prevalence decline over the past decade may result from improved intensive farming practices and better herd management strategies that reduce environmental contamination. Furthermore, enhanced parasite control measures, particularly routine deworming protocols, may have also played a significant role. These combined factors have likely contributed to the reduced transmission dynamics observed in the current study.

The high genetic diversity of the ITS gene enables extensive genotype classification of *E. bieneusi*. To date, 79 distinct genotypes have been identified in sheep and 49 in goats worldwide (Cheng et al., 2024a). In the present study, seven known genotypes were detected from 127 *E. bieneusi*-positive samples based on the ITS gene locus in domestic small ruminants, including COS-I, BEB6, CHG1, CHG3, CHS7, CHS8, and J, with BEB6 (*n* = 67) as the dominant genotype. This phenomenon has also been reported in domestic small ruminants in previous studies (Chen et al., 2018; Cheng et al., 2024a; Qin et al., 2022). Phylogenetic analyses classify *E. bieneusi* genotypes into 11 distinct groups, with Groups 1 and 2 containing genotypes demonstrating zoonotic potential and cross-species transmission capacity (Shen et al., 2020). All seven genotypes identified in the present study clustered within Group 2, aligning with observations from climatically similar provinces (Qi et al., 2019; Ye et al., 2015). Although Group 2 is usually regarded as ruminant-adapted, and its public-health relevance is considered lower than that of Group 1, BEB6 has been reported in both humans and animals in China, suggesting that limited cross-species transmission cannot be ruled out (Yu et al., 2019). These findings suggest that domestic small ruminants in Heilongjiang Province may act as reservoirs for *E. bieneusi* transmission, with potential implications for zoonotic risk in the region.

*Blastocystis* sp. has been recognized as a clinically significant intestinal protistan parasite with global distribution in domestic small ruminants (Rajamanikam et al., 2023). In the present study, the infection rate for *Blastocystis* sp. in Heilongjiang Province was 3.6 %, with prevalence rates of 3.3 % in sheep and 4.8 % in goats. For sheep, the prevalence observed in Heilongjiang Province appears among the lowest reported to date, and is lower than that reported for Shanxi Province (16.26 %, 80/492) (Wei et al., 2023), Inner Mongolia Autonomous Region (11.30 %, 39/345) (Zhang et al., 2023), Tibet Autonomous Region (8.55 %, 53/620) (Chang et al., 2021) and Qinghai Province (7.5 %, 57/761) (Yang et al., 2023). For goats, the *Blastocystis* sp. infection rate for Heilongjiang Province determined in the present study is lower than that reported for Inner Mongolia Autonomous Region (10.40 %, 72/692) (Zhang et al., 2023), and Tibet Autonomous Region (8.46 %, 22/260) (Chang et al., 2021). From a global perspective, the overall prevalence in the present study is similar to that reported from Iran (4.7 %, 7/150) (Shams et al., 2024), but lower than those reported for Malaysia (23.1 %, 87/376) (Rauff-Adedotun et al., 2023) and Turkey (38.2 %, 84/220) (Onder et al., 2021). While previous surveillance from Heilongjiang Province reported 5.5 % (6/109) in sheep and no infection (0/13) in goats (Wang et al., 2018), our study provides the first evidence of *Blastocystis* sp. infection in goats in Heilongjiang Province, Thereby expanding the known distribution of *Blastocystis* sp. in Northeastern China.

Molecular approaches are the gold standard for epidemiological investigations and subtype analysis of *Blastocystis* sp. (Stensvold et al., 2020). According to recent reviews and epidemiological surveys, at least 20 distinct STs have been identified in small ruminants globally (Naguib et al., 2024). Our phylogenetic analysis revealed considerable genetic diversity among *Blastocystis* sp. isolates from domestic small ruminants, identifying six STs (ST5, ST10, ST14, ST15, ST26, and ST30), all of which have been reported previously in ruminant hosts (Yang et al., 2023; Zhang et al., 2023). Previous reports indicate that humans can host ST1–ST4 and ST6–ST9, and more than 90 % of human *Blastocystis* strains belong to ST1–ST4 (Piperni et al., 2024). ST5 is common in domesticated ungulates, and ruminants are the primary reservoirs for ST10 (Stensvold et al., 2009). In our study, ST10 (*n* = 22) was the predominant subtype in domestic small ruminants in Heilongjiang Province. This result is consistent with previous reports from the climatically similar Inner Mongolia Autonomous Region (Zhang et al., 2023). While we did not detect the subtypes most frequently associated with human infections, ST10 and ST14 have occasionally been reported in humans in China (Figueiredo et al., 2024), suggesting that the zoonotic risk in Heilongjiang Province is likely low but not negligible and warrants continued molecular surveillance in animal populations in Heilongjiang Province.

Previous reports have observed a significant association between *Cryptosporidium* spp. and *G. duodenalis* co-infections in dogs, with the presence of one infection increasing the likelihood of the other (Mateusa et al., 2024). In the present study, the overall co-infection rate was 3.0 % (30/1011), with co-infections involving the two protozoan pathogens being the most common. Moreover, co-infections occurred more frequently in young animals than in adults, suggesting that developing immune systems may be more susceptible to the combined effects of multiple pathogens, which in turn appear to exacerbate the severity of diarrhea and pose a zoonotic risk to humans (Zhang et al., 2016). Moreover, from a One Health perspective, the detection of zoonotic protozoan in present study highlights potential animal-to-human transmission via direct contact, contaminated fomites, and fecal–oral exposure. Therefore, measures such as reducing stocking density, improving environmental disinfection, and strengthening routine surveillance are warranted to mitigate protozoan infections in animals.

These protozoan pathogens are widely distributed in the environment, particularly in surface water sources, where contamination by host feces may facilitate transmission to humans and animals (Xiao and Feng, 2017; Zhang et al., 2019). Nevertheless, this study has several limitations that warrant consideration. First, environmental samples were not analyzed, precluding assessment of potential transmission routes. Given the known environmental persistence of these protozoa, the lack of environmental sampling limits our understanding of how these pathogens might spread through water sources, soil, and other environmental media. Future investigations should integrate environmental surveillance to evaluate contamination risks and clarify environmental transmission dynamics. Second, the absence of high-resolution genotyping data for *Cryptosporidium* spp. isolates limits subtype characterization. High-resolution genotyping could provide more detailed insights into genetic diversity and potential sources of infection, which are crucial for understanding epidemiology and developing targeted control measures. Future studies should aim to overcome these limitations by employing advanced molecular techniques to better characterize parasite diversity and transmission pathways.

## 5 Conclusions

In summary, the findings in the present study demonstrate the prevalence, genotypic characterization, and risk factors for *Cryptosporidium* spp., *G. duodenalis*, *E. bieneusi*, and *Blastocystis* sp. in domestic small ruminants in Heilongjiang Province, Northeast China. Four *Cryptosporidium* species were detected, with *C. xiaoi* being the dominant species. Two *G. duodenalis* assemblages (E and A) were identified, with assemblage E predominating. Seven *E. bieneusi* genotypes were detected, all clustering within Group 2. Six *Blastocystis* subtypes were identified, with ST10 predominant. These findings highlight domestic small ruminants as potential reservoir hosts for intestinal protozoa of zoonotic relevance, providing valuable data for control strategies and regional zoonotic risk assessment.

## CRediT authorship contribution statement

**Meiru Hou:** Writing – original draft, Validation, Methodology. **Xuewei Liu:** Validation, Methodology. **Lu Zhou:** Validation, Methodology. **Jiawang Zhou:** Methodology, Investigation. **Yuxi Zhang:** Methodology, Investigation. **Tianshuai Ma:** Visualization, Data curation. **Hongyu Qiu:** Methodology, Data curation. **Chunren Wang:** Supervision, Funding acquisition. **Junfeng Gao:** Project administration, Funding acquisition, Conceptualization.

## Ethics statement

The research protocol was reviewed and approved by the Animal Ethics Committee of the Heilongjiang Bayi Agricultural University (Approval No. DWKJXY2023027). Permission was obtained from farmers or animal owners before stool sampling, and none of the animals were injured during the samples collection.

## Declaration of competing interest

The authors declare that they have no competing interests.

## Data Availability

Data supporting the conclusions of this article are included within the article. Representative nucleotide sequences obtained in the present study were deposited in GenBank with the accession numbers PQ192154–PQ192157 for *Cryptosporidium* spp., PQ212850–PQ212853 for *G. duodenalis*, PQ192604–PQ192610 for *E. bieneusi*, and PQ197594–PQ197599 for *Blastocystis* sp.

## Appendix A Supplementary data

Supplementary material 1: Primer sequences and reaction conditions used for nested PCR amplifications of four enteric protozoans. Image 1

Supplementary material 2: Occurrence of co-infection of four enteric protozoans in domestic small ruminants in Heilongjiang Province, Northeast China. Image 2
