# Supplementary material for: Molecular characteristics and zoonotic potential of enteric protozoans in domestic small ruminants in Heilongjiang Province, Northeast China
Source: Food Waterborne Parasitol. 2025 Oct 23;41:e00296. doi: 10.1016/j.fawpar.2025.e00296 (PMC12595128; doi:10.1016/j.fawpar.2025.e00296)
Supplement: Supplementary file 1 — Supplementary material 1: Primer sequences and reaction conditions used for nested PCR amplifications of four enteric protozoans. [file mmc1.docx]

Appendix A. Primer sequences and reaction conditions used for nested PCR amplifications of four enteric protozoans.

| Species | Loci | Primer ID | Primer Sequences (5'-3') | Fragment Length (bp) | Temperature Annealing (℃) |
| --- | --- | --- | --- | --- | --- |
| *Cryptosporidium* spp. | *SSU rRNA* gene | CS-F1 | TTCTAGAGCTAATACATGCG | ~830 | 55 |
|  |  | CS-R1 | CCCATTTCCTTCGAAACAGGA |  |  |
|  |  | CS-F2 | GGAAGGGTTGTATTTATTAGATAAAG |  |  |
|  |  | CS-R2 | CTCATAAGG TGCTGAAGGAGTA |  |  |
| *Giardia duodenalis* | *bg* gene | GD-F1 | GAGGCCGCCCTGGATCTTCGAGACGAC | ~510 | 60 |
|  |  | GD-R1 | GAACGAACGAGATCGAGGTCCG |  |  |
|  |  | GD-F2 | CTCGACGAGCTTCGTGTT |  |  |
|  |  | GD-R2 | TTCCGTRTYCAGTACAACTC |  |  |
| *Enterocytozoon bieneusi* | ITS gene | EB-F1 | GGTCATAGGGATGAAGAG | ~400 | 57 |
|  |  | EB-R1 | TTCGAGTTCTTTCGCGCTC |  |  |
|  |  | EB-F2 | GCTCTGAATATCTATGGCT |  |  |
|  |  | EB-R2 | ATCGCCGACGGATCCAAGTG |  |  |
| *Blastocystis* sp. | *SSU rRNA* gene | BS-F | GAGCTTTTTAACTGCAACAACG | ~600 | 57 |
|  |  | BS-R | ATCTGGTTGATCCTGCCAGTA |  |  |
