# Supplementary material for: Molecular characteristics and zoonotic potential of enteric protozoans in domestic small ruminants in Heilongjiang Province, Northeast China
Source: Food Waterborne Parasitol. 2025 Oct 23;41:e00296. doi: 10.1016/j.fawpar.2025.e00296 (PMC12595128; doi:10.1016/j.fawpar.2025.e00296)
Supplement: Supplementary file 2 — Supplementary material 2: Occurrence of co-infection of four enteric protozoans in domestic small ruminants in Heilongjiang Province, Northeast China. [file mmc2.docx]

Appendix B. Occurrence of co-infection of four enteric protozoans in domestic small ruminants in Heilongjiang Province, Northeast China.

| Region | Type | | Gender | | Age | |
| --- | --- | --- | --- | --- | --- | --- |
|  | Goat | Sheep | Female | Male | ≤ 1 Year | >1year |
| Daqing | BEB6 + ST10 (2), *C. xiaoi* + ST14 (1)*,* E + COS-I (1), *C. bovis* + COS-I (1), *C. xiaoi* + BEB6 (1) | *C. xiaoi* + COS-I (1), *C. ubiquitum* + BEB6 (1), E + BEB6 (1), *C. xiaoi* + ST10 (5), *C. ubiquitum* + BEB6 + ST10 (1), *C. ubiquitum +* ST10 (1), *C. xiaoi* + BEB6 + ST10 (1), *C. xiaoi* + BEB6 (2) | *C. xiaoi +* ST10 (2), *C. ubiquitum +* ST10 (2), *C. xiaoi +* ST14 (1), BEB6 + ST10 (1), *C. xiaoi* + BEB6 + ST10 (1), E + COS-I (1), *C. bovis* + COS-I (1), *C. xiaoi* + BEB6 (1) | *C. xiaoi* + COS-I (1), *C. ubiquitum* + BEB6 (1), E + BEB6 (1), *C. xiaoi +* ST10 (3), *C. xiaoi* + BEB6 (1), BEB6 + ST10 (2) | *C. xiaoi* + COS-I (1), *C. ubiquitum* + BEB6 (1), *C. xiaoi +* ST10 (5), *C. ubiquitum* + BEB6 + ST10 (1), *C. xiaoi* + BEB6 (2), BEB6 + ST10 (3), *C. ubiquitum +* ST10 (1), *C. xiaoi* + ST14 (1), *C. xiaoi* + BEB6 + ST10 (1), *C. bovis* + COS-I (1) | E + BEB6 (1), E + COS-I (1) |
| Hegang | — | E + COS-I (1), E + CHS7 (1) | E + COS-I (1), E + CHS7 (1) | — | — | E + COS-I (1), E + CHS7 (1) |
| Qiqihaer | E + CHG3 (1), CHG3 + ST14 (1) | — | E + CHG3 (1), CHG3 + ST14 (1) | — | E + CHG3 (1), CHG3 + ST14 (1) | — |
| Shuangyashan | *C. xiaoi* + BEB6 (1), BEB6 + ST26 (1) | — | *C. xiaoi* + BEB6 (1) | BEB6 + ST26 (1) | — | *C. xiaoi* + BEB6 (1), BEB6 + ST26 (1) |
| Suihua | BEB6 + ST10 (1), COS-I + ST14 (3) | — | BEB6 + ST10 (1), COS-I + ST14 (1) | COS-I + ST14 (2) | — | BEB6 + ST10 (1), COS-I + ST14 (3) |
| Yichun | — | BEB6 + ST26 (1) | — | BEB6 + ST26 (1) | — | BEB6 + ST26 (1) |
